# Alkaline Ozonation-Induced TiO_2_ Nanoscaffold on Titanium Alloy for Surface-Mediated Osteogenic Guidance

**DOI:** 10.3390/jfb17060274

**Published:** 2026-06-01

**Authors:** Mariusz Winiecki, Piotr Krawczyk, Katarzyna Reczyńska-Kolman, Iwona Pudełko-Prażuch, Elżbieta Pamuła, Marek Trzcinski

**Affiliations:** 1 Department of Constructional Materials and Biomaterials, Faculty of Materials Engineering, Kazimierz Wielki University, 30 J.K. Chodkiewicza Street, 85-064 Bydgoszcz, Poland; 2 Institute of Chemistry and Technical Electrochemistry, Faculty of Chemical Technology, Poznan University of Technology, 4 Berdychowo Street, 61-131 Poznan, Poland; 3 Department of Biomaterials and Composites, Faculty of Materials Science and Ceramics, AGH University of Krakow, 30 A. Mickiewicza Avenue, 30-059 Krakow, Poland; kmr@agh.edu.pl (K.R.-K.); ipudelko@agh.edu.pl (I.P.-P.); epamula@agh.edu.pl (E.P.); 4 Division of Surface Science, Faculty of Chemical Technology and Engineering, Bydgoszcz University of Science and Technology, 7 Prof. S. Kaliskiego Avenue, 85-796 Bydgoszcz, Poland; marek.trzcinski@pbs.edu.pl

**Keywords:** titanium alloy, surface modification, alkaline ozonation, advanced oxidation, TiO_2_ nanonetwork, nanoscaffold, osteogenic differentiation

## Abstract

Numerous surface modification strategies, particularly nanoengineering approaches, have been explored to tailor the physicochemical and topographical properties of titanium surfaces in order to enhance osteogenic responses at the implant interface. In this study, we propose an alkaline ozonation strategy as a novel approach to generate nanostructured TiO_2_ layers on Ti-6Al-4V alloy surfaces. Titanium discs were treated in a 6 M KOH solution under continuous bubbling of ozone, allowing the formation of reactive oxygen species (ROS) responsible for oxidative surface restructuring. Scanning electron microscopy (SEM) revealed the formation of a homogeneous three-dimensional TiO_2_ nanonetwork composed of intertwined nanofibers. X-ray photoelectron spectroscopy (XPS) confirmed the oxidative reconstruction of the Ti alloy surface. The fraction of Ti^4+^ species characteristic of TiO_2_ increased markedly from 44.2 at% to 92.2 at%, accompanied by a strong reduction in Ti^0^ (from 40.2 at% to 5.8 at%) and Ti^3+^ (from 15.7 at% to 2.1 at%). Concomitantly, lattice oxygen associated with Ti–O–Ti bonding increased from 48 at% to 78 at% as deduced from the O 1s signal, while the surface carbon content decreased from 48 at% to 18 at%. The modification induced a pronounced increase in surface hydrophilicity, with the water contact angle decreasing from 85° to 32° and the surface free energy increasing from 40.8 mJ/m^2^ to 69.8 mJ/m^2^. In vitro studies demonstrated good cytocompatibility and enhanced osteogenic differentiation of human mesenchymal stem cells, with twice as much alkaline phosphatase activity after 14 days and mineralization of the extracellular matrix after 28 days than those on TCPS, and also significantly higher than those on the nonmodified Ti alloy control. These findings indicate that the generated three-dimensional TiO_2_ nanonetwork acts as a surface-confined nanoscaffold providing nanoscale cues that promote osteogenic cell responses on titanium implant surfaces.

## 1. Introduction

Titanium (Ti) and its alloys have been widely used in hard tissue surgery for over six decades due to their favorable mechanical properties and excellent biocompatibility, making them benchmark materials for orthopedic and dental implants [1,2,3]. However, their native oxide layer on Ti is essentially bioinert and displays limited intrinsic bioactivity, which can retard early bone healing at the bone-implant interface [4]. Consequently, numerous surface modification strategies have been developed to alter the physicochemical surface properties of Ti-based materials, including composition, surface free energy, wettability, and topography, thus improving osseointegration and improving their biological performance [3,5,6,7].

Cellular responses at the bone-implant interface are governed by both micro- and nanoscale surface features; accordingly, considerable research efforts have been directed toward nanoscale surface engineering to promote osteogenic activity and improve interfacial bone formation [8,9,10,11,12]. Nanostructured architectures, for example, fibers, spheres, sheets, hollow tubes, or interconnected networks, can serve as surface-confined nanoscaffolds that modulate protein adsorption and cell fate decisions [11,12]. A range of chemical and electrochemical strategies—including alkaline-based treatments (alkali soaking/alkaline hydrothermal processing), conventional anodization, and micro-arc oxidation (MAO)—have been employed to fabricate nanostructured TiO_2_ architectures that enhance osteogenic cell responses and support bone formation in vitro and in vivo [13,14,15,16,17,18,19,20,21,22].

Alkaline-based chemical treatments (e.g., alkali soaking or alkaline hydrothermal processing) typically produce nanoscale networks or nanosheet architectures that act as bioactive templates for apatite nucleation [23,24,25] and have been shown to promote early osteogenic markers (e.g., ALP) [24,25] and stem cell osteogenic differentiation [26]. Interconnected TiO_2_ nanonetwork architectures on titanium generated by electrochemical anodization have been demonstrated to enhance human bone marrow mesenchymal stem cells (hBMSCs) towards the osteogenic differentiation pathway and convert the surface of Ti from bioinert to bioactive [27,28,29].

Ozone (O_3_), a strong oxidizing agent, is recognized for its ability to modify, regenerate, and activate the surface of different materials [30,31]. Ozone treatment, as an example of a highly oxidizing process, in the vast majority of cases introduces oxygen into the surface of modified materials, most often in the form of oxygen groups. However, advanced oxidation can also cause structural changes in the materials under investigation. An example of this is carbon materials, which, due to the intensive ozone oxidation, alter their porous structure, thus changing their surface area [30,31,32]. The mechanism of the modification mentioned is based on the decomposition of the carbon structure as a consequence of its reaction with ozone. To date, only a limited number of studies have investigated gaseous ozone treatment of titanium surfaces [33,34,35]; nevertheless, such treatment has been reported to chemically activate the surface without altering its original topography [33,34], while promoting mesenchymal stem cell proliferation and osteogenic differentiation [35]. Under alkaline conditions, ozone undergoes hydroxide-initiated radical chain decomposition, leading to the in situ formation of reactive oxygen species (ROS), predominantly hydroxyl radicals (HO^•^), which are considered the main oxidation agent [36,37]. Within the possible scenario, further reaction pathways through generated reactive oxygen species containing oxygen (O_3_^•−^, O_2_^•−^, HO^•^, HO_2_^•^, HO_3_^•^, HO_4_^•^), accompanied by further ozone consumption, diffuse to the surface of the material immersed in a solution, resulting in its progressive oxidation [38]. These highly reactive species can attack surface bonds, promoting oxidative etching and gradual restructuring of the outermost layer of the material, which may result in the formation of nanoporous or nanostructured oxide layers.

In this study, we propose an alkaline ozonation strategy to fabricate a TiO_2_ nanoscaffold on Ti alloy surfaces to promote surface-mediated osteogenic responses. To our knowledge, the effect of ROS generated during alkaline ozone decomposition on the formation of osteoinductive TiO_2_ nanoarchitectures has not been elucidated so far.

## 2. Materials and Methods

### 2.1. Sample Preparation

Disc-shaped specimens measuring 8 mm in diameter and 2 mm in thickness were fabricated from a Ti-6Al-4V (Grade 5) alloy rod (Bibus Metals, Dąbrowa, Poland). The samples were ground manually using silicon carbide abrasive papers with grit sizes of 600, 1200, and 2000 (Buehler, Lake Bluff, IL, USA). Subsequently, the discs were ultrasonically cleaned in acetone and distilled water for 10 min in each medium and then dried under ambient conditions. These untreated specimens served as reference samples and were designated as Ti-ref. Prior to surface modification, the discs were etched in 60% sulfuric acid, rinsed with ultrapure water, and air-dried at room temperature for 24 h. The prepared samples were then immersed in an electrolyte consisting of a 6 M aqueous potassium hydroxide (KOH) solution (Sigma-Aldrich, St. Louis, MO, USA). The modification process was carried out in a glass reactor filled with the KOH electrolyte. Ozone was continuously introduced into the reactor at a flow rate of 0.5 dm^3^/min, and the treatment was performed for 120 min at room temperature. Ozone was generated using air supplied to the ozone generator. Samples subjected to ozone-assisted treatment in the liquid phase were labeled as Ti-O_3_.

### 2.2. Sample Characterization

The surface morphology of the specimens was characterized using a scanning electron microscope (SEM) (Hitachi SU8010; Hitachi High-Technologies Co., Tokyo, Japan). X-ray photoelectron spectroscopy (XPS) analyses were conducted with a VG-Scienta R3000 spectrometer (VG-Scienta, Uppsala, Sweden). The excitation source was an RS 40B1 lamp (Prevac Sp. z o.o., Rogów, Poland) emitting Al Kα radiation (1486.6 eV). Spectra were acquired from an analysis area of 4 mm × 0.16 mm, while photoelectrons were detected at a take-off angle of 90°. High-resolution spectra were recorded for the Ti 2p, C 1s, and O 1s regions using an energy increment of 100 meV. All XPS spectra were charge-referenced to the adventitious carbon C 1s peak at 284.8 eV. Throughout the measurements, the pressure inside the analysis chamber remained below 5 × 10^−10^ mbar. Spectral fitting and peak deconvolution were carried out with CasaXPS software (version 2.3.16, Casa Software Ltd., Teignmouth, UK) applying a Shirley-type background model.

The physicochemical characteristics of the samples were evaluated through wettability and surface free energy (SFE) measurements. Contact angle analyses were performed using a DSA 25 goniometer (A. KRÜSS Optronic GmbH, Hamburg, Germany). Wettability was determined by the sessile drop technique employing 1 μL droplets of deionized water. For the determination of surface free energy, contact angle measurements were conducted using diiodomethane (Sigma-Aldrich, St. Louis, MO, USA) under the same experimental conditions as those applied for water. The SFE values were subsequently calculated according to the Owens–Wendt method. All measurements were carried out at ambient temperature (20 °C).

### 2.3. In Vitro Cytocompatibility

In vitro cytocompatibility of the samples was tested in contact with MG-63 osteoblast-like cells. Cells (5000 cells per sample) were seeded on tested samples and on tissue culture polystyrene (TCPS) as control, and cultured in Minimum Essential Medium (MEM), supplemented with fetal bovine serum (FBS, 10%), a mixture of antibiotics (penicillin/streptomycin, P/S, 1%), sodium pyruvate (0.1%) and non-essential amino acids (0.1%) (all from PAN-Biotech GmbH, Aidenbach, Germany) for up to 7 days at 37 °C in a 5.0% CO_2_ atmosphere. After 1, 3, and 7 days, live/dead staining and AlamarBlue assay were performed. For the AlamarBlue metabolic assay, a 10% solution of AlamarBlue (resazurin sodium salt, Sigma-Aldrich, St. Louis, MO, USA) in an MEM was prepared, added into each well, and incubated for 3 h. Subsequently, 150 µL of medium was transferred in triplicate to a black 96-well plate and the fluorescence was measured (*λ*_ex_ = 544 nm and *λ*_em_ = 590 nm, FluoStar Omega, BMG Labtech, Ortenberg, Germany). The resazurin reduction was then calculated using the following formula:

(1) $$\mathrm{AlamarBlue}\;\mathrm{reduction}\;[\%]\;=\;\frac{R_{i}-R_{0}}{R_{100}-R_{0}}\times 100,$$

where

*R_i_*—reduction in sample.
*R*_0_—reduction in MEM with AlamrBlue reagent (0% reduction).
*R*_100_—reduction in autoclaved MEM with AlamrBlue reagent (100% reduction).

For live/dead staining, calcein AM and propidium iodide (both 0.1%) were mixed with phosphate-buffered saline (PBS). Then, the prepared solution was added to each well and incubated for 20 min in the dark. After that, pictures of live and dead cells were taken with the use of a fluorescent microscope (ZEISS Axiovert 40 CFL with metal halide illuminator, Oberkochen, Germany).

### 2.4. Osteogenic Differentiation

For differentiation studies, all tested samples were placed in a 48-well plate. Human bone marrow mesenchymal stem cells (hBMSCs, PromoCell, passage 10) were seeded at 40,000 cells/sample initially in 50 µL of growth medium (PromoCell growth medium supplemented with 1% P/S) and allowed to sediment and adhere to the surface of the sample for 30 min. Then, 0.45 mL of growth medium was added to each well and the culture was carried out for up to 28 days (cell culture medium was gently replaced every 2–3 days). TCPS was used as a control substrate.

Early osteogenic differentiation was evaluated using alkaline phosphatase (ALP) activity assessment on days 7 and 14. The samples were transferred to a new 48-well plate and washed with 0.5 mL of PBS. Cells were then lysed with 200 µL of 1% solution of Triton X-100 (Sigma-Aldrich) for 50 min on a horizontal shaker at 100 rpm (SO-200 Vortex Mixer, Labnet International Inc., Edison, NJ, USA). 25 µL of cell lysate was transferred in triplicate to a transparent 96-well plate and mixed with 125 µL of the ALP working solution, obtained by dissolving 20 mg of p-nitrophenylphosphate (pNPP, Sigma-Aldrich, St. Louis, MO, USA) in 20 mL of ALP buffer containing 0.1 M Tris, 0.1 M NaCl and 5 mM MgCl_2_ (Avantor Performance Materials, Gliwice, Poland). The plate was incubated at 37 °C for 30 min, then 63 µL of 1 M NaOH was added to stop the reaction. The absorbance was measured at 405 nm using a microplate reader (FLUOstar Omega, BMG Labtech, Ortenberg, Germany). The BCA assay kit (Bicinchoninic Acid Protein Assay Kit, Sigma-Aldrich) was used to determine the total protein content in the samples, and the ALP activity was normalized to protein levels.

After 21 and 28 days of the culture, the samples were transferred into a new 48-well plate and washed 2 times with 0.5 mL of PBS. Cells were fixed with 4% formaldehyde (Sigma-Aldrich, St. Louis, MO, USA) for 20 min, washed again with water and then stained with 0.5 mL of 40 mM Alizarin Red (Sigma-Aldrich, St. Louis, MO, USA) solution in Milli-Q water (pH = 4.2 adjusted with 1 M HCl, Avantor Performance Materials) for 5 min. The cells were then washed with Milli-Q water and visualized using an optical microscope (Keyence VHX-7000, KEYENCE, Mechelen, Belgium). The amount of bound Alizarin Red was measured after extraction of the dye with 0.5 mL of 10% *w*/*v* cetylpyridinium chloride (Carl Roth GmbH + Co., Karlsruhe, Germany) for 30 min. An amount of 100 µL of the extracts were transferred in triplicate to a transparent 96-well plate and the absorbance was measured at 550 nm (FluoStar Omega, BMG Labtech, Ortenberg, Germany).

### 2.5. Statistics

All the quantitative data are shown as average ± standard deviation.

One-way analysis of variance (ANOVA), followed by Tukey post hoc test with OriginLab software (version 2022 SR1, OriginLab Corporation, Northampton, MA, USA), was used to determine statistical differences; *p* < 0.05 were considered statistically significant.

## 3. Results and Discussion

**Figure 1:**
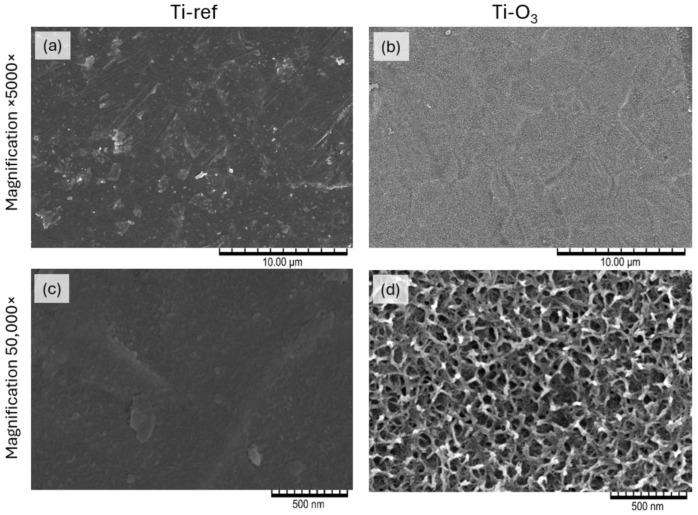
SEM images of Ti-ref (**a**,**c**) and Ti-O_3_ (**b**,**d**); acceleration voltage: 5.0 kV; magnification: 5000× (**a**,**b**), 50,000× (**c**,**d**).

Figure 1 shows the surface microstructure of the Ti-ref and Ti-O_3_ discs. At lower magnification (Figure 1a), the Ti-ref discs exhibit a relatively smooth and flat surface with numerous grooves resulting from prior polishing with abrasive paper. On the contrary, at the same magnification (Figure 1b), the surface of the Ti-O_3_ disc displays a slightly corrugated morphology with etching-induced pits and a fine mesh-like structure that is homogeneously distributed across the surface. At higher magnification, compared to the smooth and flat surface of the Ti-ref discs (Figure 1c), the Ti-O_3_ discs reveal a topography consisting of a network of intertwined nanofibers that form a three-dimensional, multilayered nanostructure (Figure 1d). These observations indicate that ozone treatment in a liquid environment of KOH solution promotes the growth of a nanostructured surface layer on titanium alloy surfaces. It should be emphasized, that during the ozone treatment of the investigated sample, radical compounds generated during the reaction of ozone with hydroxyl ions play a key role in surface modification. ROS diversity and oxidizing strength guarantee effective oxidation of the surface and its nanostructurization.

**Figure 2:**
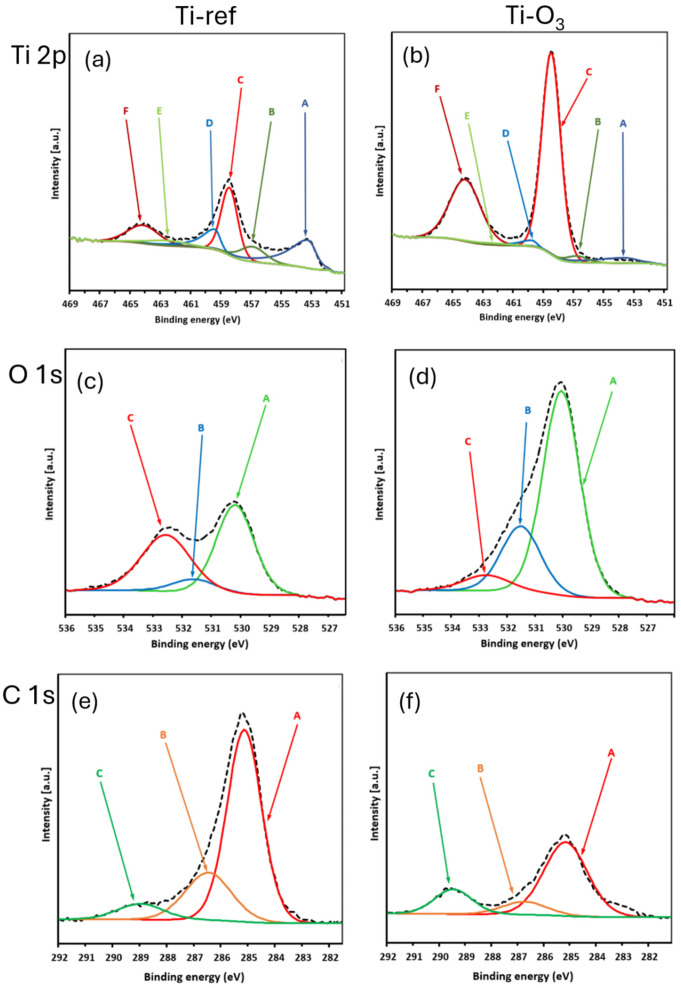
XPS spectra of Ti 2p region (**a**,**b**), O 1s region (**c**,**d**) and C 1s region (**e**,**f**), recorded for Ti-ref (**a**,**c**,**e**) and Ti-O_3_ (**b**,**d**,**f**). Assignment of peaks is shown in Table 1.

The chemical composition of the surfaces of the Ti-ref and Ti-O_3_ discs, obtained from XPS measurements, is listed in Table 1, while Figure 2 depicts the deconvoluted XPS spectra of the Ti 2p region (Figure 2a,b), O 1s region (Figure 2c,d), and C 1s region (Figure 2e,f), where Δ is a spin–orbit energy splitting value between Ti 2p_3/2_ and Ti 2p_1/2_ levels.

The surface of the investigated samples consists of titanium, oxygen, and carbon. A significant increase in Ti and O content and a marked decrease in C content (Table 1) is observed due to ozonation, confirming the oxidative character of the performed surface treatment. Specifically, such a treatment induces a pronounced increase in surface oxygen from 31.9 at% to 64.7 at%, a moderate increase in Ti from 19.7 at% to 24.3 at%, and a substantial reduction in adventitious carbon from 48.4 at% to 11.0 at%.

The Ti 2p spectra of the Ti-ref and Ti-O_3_ discs differ substantially (Figure 2a,b). Both spectra decompose into three levels (doublets) corresponding to three oxidation states of Ti, with Ti 2p_3/2_ peaks at binding energy of 453.6 eV (peak A), 456.8 eV (peak B) and 458.5 eV (peak C) attributed to Ti^0^, Ti^3+^ in Ti_2_O_3_ and Ti^4+^ in TiO_2_, respectively [39,40]. The remaining peaks (D = 459.8 eV; E = 462.5 eV; F = 464.2 eV) correspond to the Ti 2p_1/2_ sublevels of the Ti^0^, Ti^3+^, and Ti^4+^ components, respectively. Deconvolution of the Ti 2p spectra reveals that the Ti-ref surface consists of a mixture of metallic Ti, dititanium trioxide (Ti^3+^), and Ti^4+^, whereas in the Ti-O_3_ sample the Ti signal is dominated by Ti^4+^ (TiO_2_), increasing from 8.7 at% (44.2% of total Ti content) to 22.4 at% (92.2% of total Ti content), with only traces of Ti^0^ (decreasing from 7.9 at% constituting 40.2% of total Ti content to 1.4 at%, i.e., 5.8% of total Ti content) and Ti^3+^ (decreasing from 3.1 at%, i.e., 15.7% of total Ti content, to 0.5 at%, i.e., 2.1% of total Ti content).

The O 1s spectra of both investigated discs (Figure 2c,d) comprise three peaks located at binding energies of 530.1 eV (lattice oxygen in TiO_2_), 531.6 eV (hydroxyl groups) and 532.8 eV (adsorbed water) [40,41,42]. A strong increase in lattice O (Ti–O–Ti) from 15.3 at% (48.0% of total O content) in Ti-ref discs to 50.5 at% (78.0% of total O content) in Ti-O_3_ discs, increased surface hydroxylation from 3.0 at% (9.4% of total O content) in Ti-ref discs to 13.8 at% (21.3% of total O content) in Ti-O_3_ discs, and substantial removal of loosely bound or adsorbed oxygen species, decreasing from 13.6 at% (42.6% of total O content) in Ti-ref discs to 0.5 at% (0.8% of total O content) in Ti-O_3_ discs, confirms the transformation observed in the Ti 2p spectra.

The deconvolution of C 1s spectra (Figure 2e,f) reveals three components corresponding to aliphatic carbon C–C and C–H (285.2 eV), C–O (286.8 eV), and O–C=O (289.5 eV) [40,42]. Comparison of the spectra indicates that the surface carbon contamination is substantially removed due to oxidation: C–C decreases from 33.4 at% in Ti-ref discs to 6.2 at% in Ti-O_3_, C–O decreases from 11.7 at% in Ti-ref discs to 2.1 at% in Ti-O_3_, and O–C=O decreases to a much lesser extent from 3.3 at% to 2.6 at%. The remaining carbon on the surface of Ti-O_3_ is enriched in oxidized forms, with the relative O–C=O share of total carbon increasing from 6.9% to 24.1%. This behaviour can be explained, on the one hand, by the effects of some transformation, but it is more likely that during advanced oxidation, carbon species degrade, resulting in the formation of CO and CO_2_, which are further removed from the oxidized surface. It seems that C–C, C–H and C–O bonds are the most susceptible to oxidation; therefore, the loss of these bonds is observed to the greatest extent. The effects described above may confirm the possible structural changes within the modified material as a result of its reaction with the products of the reaction of ozone with OH^−^ ions of the ozonated solution.

**Figure 3:**
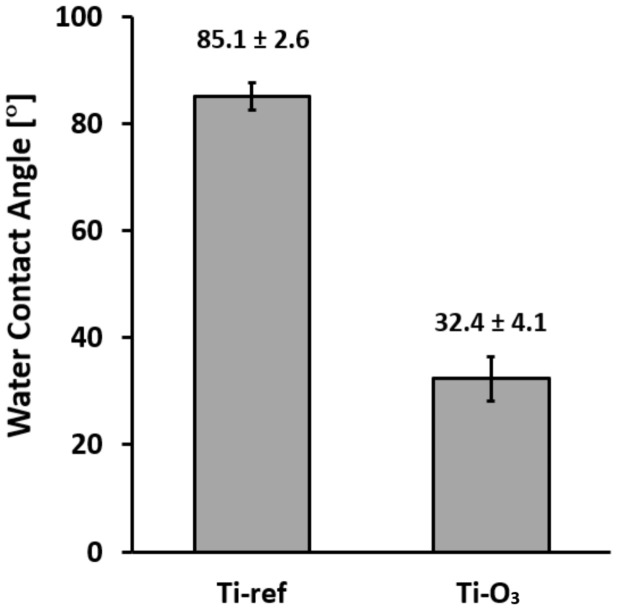
Water contact angle of Ti-ref and Ti-O_3_. Average ± standard deviation, *n* = 6. Statistically significant differences between both samples were found at *p* ≤ 0.001.

The average value of the measured contact angle for the tested specimens is shown in Figure 3. The surface free energy of the examined samples is given in Table 2. The surface of Ti-ref exhibits a contact angle of 85.1° ± 2.6°, indicating its more hydrophobic character. Following ozone treatment, a marked decrease in water contact angle to 32.4° ± 4.1° indicates a transition to a strongly hydrophilic surface. A similar effect of the performed modification is observed in the case of surface free energy.

The observed increase in total surface free energy from 40.8 ± 1.1 mJ/m^2^ to 69.8 ± 0.7 mJ/m^2^ is driven primarily by the polar component, which increased from 2.2 ± 0.8 mJ/m^2^ to 25.6 ± 3.7 mJ/m^2^. This increase is directly correlated with the XPS results, which revealed a substantial enrichment in Ti^4+^ species, and a marked rise in the lattice O^2−^ and hydroxyl-related components as clearly observed in the O 1s region, which confirms the formation of thicker, more stoichiometric and more hydroxylated TiOu_2_. The latter is a source of polar groups (–OH, Ti–O) that increase the polar component of the SFE and reduce the water contact angle. Simultaneously, the surface carbon content decreased significantly, indicating the removal of adventitious hydrocarbon contamination and the exposure of polar oxide sites.

**Figure 4:**
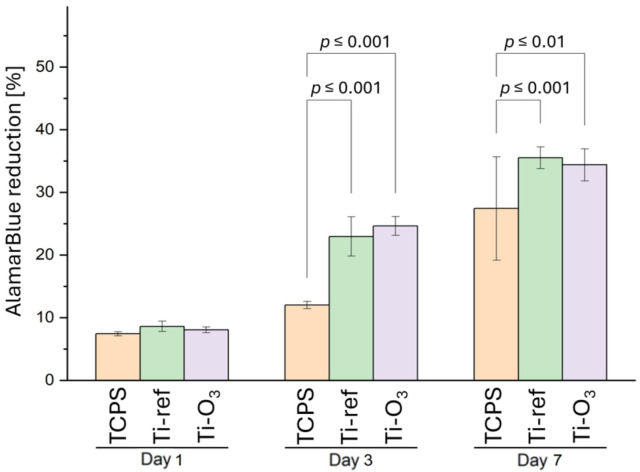
Viability of MG-63 cells cultured for 1, 3 and 7 days on Ti-ref, Ti-O_3_ and on control TCPS assessed by AlamarBlue reduction test. Statistically significant differences between samples at each time point, at *p* ≤ 0.01 and *p* ≤ 0.001 according to ANOVA followed by Tukey post hoc test.

**Figure 5:**
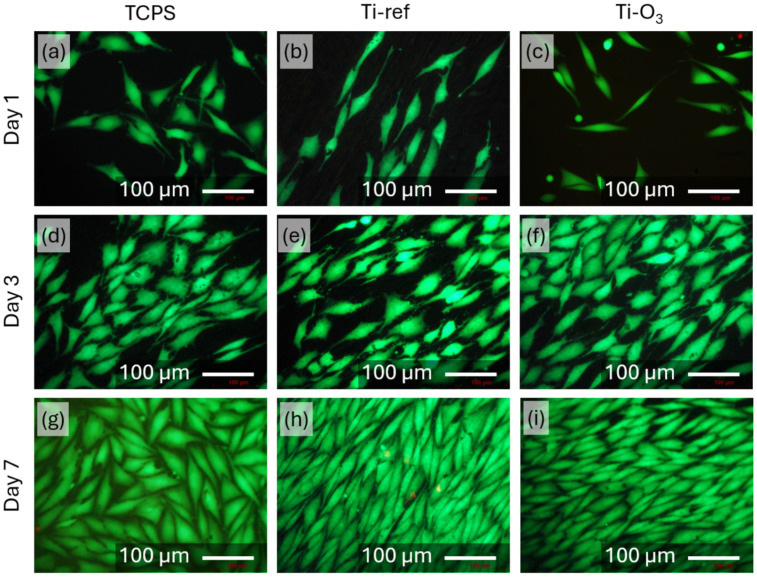
Live/dead staining of MG-63 osteoblast-like cells cultured on TCPS (**a**,**d**,**g**), Ti-ref (**b**,**e**,**h**), and Ti-O_3_ discs (**c**,**f**,**i**) for 1 day (**a**–**c**), 3 days (**d**–**f**) and 7 days (**g**–**i**). Live cells are stained green; red nuclei of dead or dying cells are stained red. Scale bar: 100 μm.

MG-63 cells were cultured on the investigated samples for 1, 3 and 7 days. The AlamarBlue assay and live/dead staining were performed after each of the predetermined days of culture. During the experiment, the cells proliferated properly on each surface. Resazurin reduction for all samples after the first day of culture was on the same level, and no statistically significant differences were observed (Figure 4). After day 3 and day 7, the results of metabolic activity were better for both Ti-ref and Ti-O_3_, compared to TCPS; however, statistical analysis revealed no significant differences between raw and modified Ti samples. The results of live/dead staining (Figure 5) correspond to the results of the AlamarBlue assay. The number of living cells were increasing, and more cells were visible on each surface after each day of culture.

**Figure 6:**
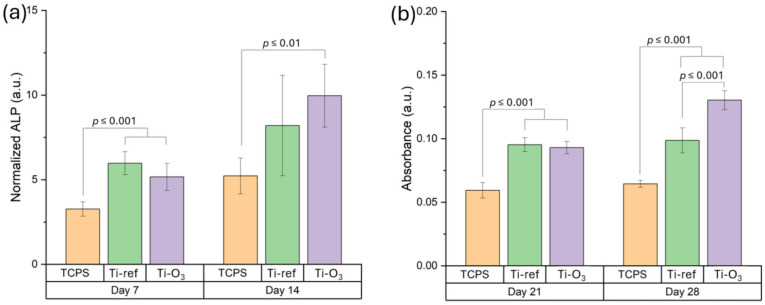
Osteogenic differentiation of hBMSCs cultured on TCPS, Ti-ref and Ti-O_3_: quantification of ALP after 7 and 14 days of culture (**a**), and quantification of Alizarin Red after 21 and 28 days of culture (**b**). Statistically significant differences between samples at *p* ≤ 0.01 and *p* ≤ 0.001 according to ANOVA followed by Tukey post hoc test.

The potential of the materials examined to induce osteogenic differentiation of hBMSCs was assessed in growth medium. As indicated by the ALP activity measurements (Figure 6) after 7 and 14 days of culture, early osteogenic differentiation was observed in both Ti-ref and Ti-O_3_ samples. On day 7, there was no statistically significant difference between Ti-ref and Ti-O_3_; however, on day 14, the highest average ALP level was observed for Ti-O_3_, and it was approximately two times higher than in the cells growing on a control substrate (TCPS).

**Figure 7:**
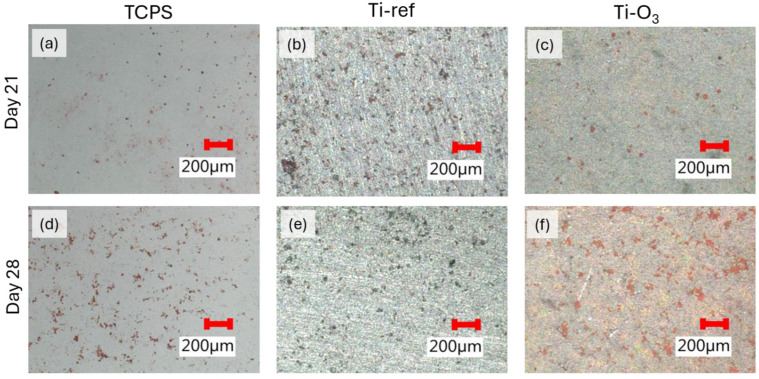
Representative images of hBMSCs cultured on TCPS (**a**,**d**), Ti-ref (**b**,**e**) and Ti-O_3_ (**c**,**f**), stained with Alizarin Red after 21 days (**a**–**c**) and 28 days (**d**–**e**).

Alizarin Red staining (Figure 7) was used to visualize the presence of calcium deposits within the extracellular matrix produced by hBMSCs. After 21 days, mineralization was similar on both examined samples. Interestingly, after 28 days of culture, a much higher amount of calcium deposits were present on the surface of Ti-O_3_ samples as compared to Ti-ref. Quantitative analysis confirmed that the amount of calcium deposits was two times higher than on TCPS and 1.3 times higher than on the unmodified Ti surface (Ti-ref) (Figure 6b). It should be emphasized that all experiments were conducted exclusively in growth medium, so the observed osteogenic differentiation was induced by surface properties of the materials only, without any external stimulation originating from, e.g., substances present in the cell culture medium.

Our observations are in line with the findings reported in the literature. Yang et al. [28] also observed that anodization of the Ti surface, leading to the formation of a three-dimensional porous network on the surface of Ti, improved mineralization of the extracellular matrix of hBMSCs. Gene expression analyses confirmed that Runx2 and Osterix mRNA levels were significantly higher in the cells cultured for 3 and 5 days on anodized Ti surfaces compared to polished Ti samples. A more pronounced differentiation of hBMSCs growing on the modified surface was also confirmed by a higher expression of late mineralization markers, such as bone sialoprotein, osteocalcin and collagen type I, after 14 and 21 days of culture. Another study found that similar surface modifications can also support the growth and differentiation of hBMSCs cells in vivo in a murine model [27].

## 4. Conclusions

In this study, alkaline ozonation was used to generate a three-dimensional TiO_2_ nanoscaffold on Ti-6Al-4V surfaces and the physicochemical and biological consequences of this modification were investigated. The surface of the Ti-6Al-4V titanium alloy was modified by exposing it to ozone bubbled through a 6 M KOH solution, thus promoting the formation of ROS responsible for surface oxidation and nanostructure formation. The resulting surfaces were characterized in terms of morphology, chemical composition, and wettability, and the biological response of the modified surfaces was subsequently evaluated in vitro.

SEM revealed the formation of a three-dimensional TiO_2_ nanonetwork on the surface of Ti-6Al-4V. XPS demonstrated a pronounced increase in Ti^4+^ species characteristic of TiO_2_, with their contribution rising from 44.2% to 92.2% of the total titanium content, accompanied by a strong reduction in Ti^0^ (40.2% to 5.8%) and Ti^3+^ species (15.7% to 2.1%). Consistently, the contribution of lattice oxygen associated with Ti–O–Ti bonding increased from 48% to 78%, as deduced from O 1s signal, while the surface carbon content decreased from 48% to 18%, confirming the oxidative reconstruction of the native oxide layer towards a more stoichiometric and partially hydroxylated TiO_2_ surface. Water contact angle decreased from approximately 85° for the reference titanium surface to approximately 32° after alkaline ozonation, while the total surface free energy increased from 40.8 to 69.8 mJ/m^2^. In particular, this increase was primarily associated with a pronounced rise in the polar component (from 2.2 ± 0.8 to 25.6 ± 3.7 mJ/m^2^), which is consistent with the formation of polar surface functionalities, such as hydroxyl groups, in the modified TiO_2_ layer.

The biological studies further demonstrated that the Ti-O_3_ surfaces maintain cyto-compatibility while promoting osteogenic activity. MG-63 osteoblast-like cells exhibited viability and proliferation comparable to those on the Ti-ref and TCPS controls, indicating the absence of cytotoxic effects. Importantly, hBMSCs cultured on Ti-O_3_ surfaces showed enhanced osteogenic differentiation: ALP activity after 14 days and extracellular matrix mineralization after 28 days on Ti-O_3_ were approximately twice that on TCPS and significantly higher than that on nonmodified titanium alloy. It is worth mentioning that these results were obtained under standard cell growth conditions without osteogenic supplements, indicating the predominant role of the performed modification.

In summary, alkaline ozonation of Ti-6Al-4V yields a three-dimensional TiO_2_ nanonetwork providing scaffold-like nanoscale cues that support cell adhesion and promote hBMSCs osteogenic differentiation under standard culture conditions. Taken together, these findings indicate that alkaline ozonation represents a simple and scalable strategy for generating bioactive nanostructured scaffolds on titanium alloy surfaces, with promising applications in orthopedic and dental implants.

## Figures and Tables

**Table 1:** XPS surface chemical composition of Ti-ref and the Ti-O_3_ samples with assignment of peaks in Ti 2p, O 1s and C 1s regions.

Spectrum	Peak	Ti-ref				Ti-O_3_				Peak Assignment
		Ti 2p_3/2_	**Δ**	Concentration	Total Concentration	Ti 2p_3/2_	**Δ**	Concentration	Total Concentration	
		**eV**	**eV**	**at%**	**at%**	**eV**	**eV**	**at%**	**at%**	
Ti 2p	A, D	453.6	6.2	7.9	19.7	453.6	6.2	1.4	24.3	Ti^0^
	B, E	456.8	5.7	3.1		456.8	5.7	0.5		Ti^3+^ (Ti_2_O_3_)
	C, F	458.5	5.7	8.7		458.5	5.7	22.4		Ti^4+^ (TiO_2_)
O 1s	A	530.1		15.3	31.9	530.1		50.5	64.7	O^2−^ in TiO_2_
	B	531.6		3.0		531.6		13.8		OH^−^
	C	532.8		13.6		532.8		0.5		H_2_O
C 1s	A	285.2		33.4	48.4	285.2		6.2	11.0	C–C/C–H
	B	286.8		11.7		286.8		2.1		C–O/C–OH
	C	289.5		3.3		289.5		2.6		O–C=O

**Table 2:** Surface free energy (SFE) of Ti-ref and Ti-O_3_ samples, where *σ* is total SFE, *σ^D^* is dispersive part of SFE and *σ^P^* is polar part of SFE.

Sample	*σ* [mJ/m^2^]	*σ^D^* [mJ/m^2^]	*σ^P^* [mJ/m^2^]
Ti-ref	40.8 ± 1.1	38.6 ± 1.4	2.2 ± 0.8
Ti-O_3_	69.8 ± 0.7	44.2 ± 3.6	25.6 ± 3.7

## Data Availability

The data presented in this study are available upon request from the corresponding author.
